# The extent and nature of supermarket own brand foods in Australia: study protocol for describing the contribution of selected products to the healthfulness of food environments

**DOI:** 10.1186/s12937-018-0404-4

**Published:** 2018-10-25

**Authors:** Claire Elizabeth Pulker, Georgina S. A. Trapp, Frances Foulkes-Taylor, Jane Anne Scott, Christina Mary Pollard

**Affiliations:** 1 School of Public Health, Curtin University, Kent Street, GPO Box U1987, Perth, Western Australia 6845 Australia; 2 Telethon Kids Institute, The University of Western Australia, PO Box 855, West Perth, Western Australia 6872 Australia; 3 School of Population and Global Health, The University of Western Australia, 35 Stirling Highway, Crawley, Western Australia 6009 Australia; 4 East Metropolitan Health Service, Kirkman House, 20 Murray Street, East Perth, Western Australia 6004 Australia; 5 Curtin University, School of Public Health, Kent Street, Bentley, Western Australia 6012 Australia

**Keywords:** Supermarket, Supermarket own brand, Nutritional quality, Marketing, Retail food environment, Food processing

## Abstract

**Background:**

While public health experts have identified food environments as a driver of poor diet, they also hold great potential to reduce obesity, non-communicable diseases, and their inequalities. Supermarkets are the dominant retail food environment in many developed countries including Australia. The contribution of supermarket own brands to the healthfulness of retail food environments has not yet been explored. The aim of this protocol is to describe the methods developed to examine the availability, nutritional quality, price, placement and promotion of supermarket own brand foods within Australian supermarkets.

**Methods:**

Photographic audits of all supermarket own brand foods present in three major food retail outlets were conducted. Two researchers conducted the supermarket audits in Perth, Western Australia in February 2017. Photographs showing the location of the in-store product display, location of products on shelves, use of display materials, and front-of-pack and shelf-edge labels were taken for each supermarket own brand food present. An electronic filing system was established for photographs from each of the supermarkets and an Excel database constructed. The following data were extracted from the photographs: front-of-pack product information (e.g. product and brand name, pack weight); packaging and label design attributes (e.g. country of origin; marketing techniques conveying value for money and convenience); shelf-edge label price and promotion information; placement and prominence of each product; and nutrition and health information (including supplementary nutrition information, nutrition and health claims, and marketing statements and claims). Nutritional quality of each product was assessed using the principles of the Australian Guide to Healthy Eating, the NOVA classification of level of food processing, and the Health Star Rating score displayed on the front-of-pack.

**Discussion:**

Approximately 20,000 photographic images were collected for 3940 supermarket own brand foods present in this audit: 1812 in the Woolworths store, 1731 in the Coles store, and 397 in the IGA store. Analysis of findings will enable researchers to identify opportunities for interventions to improve the contribution of supermarket own brands to healthful retail food environments. This protocol is unique as it aims to investigate all aspects of retail food environments and address the contribution of supermarket own brands.

**Electronic supplementary material:**

The online version of this article (10.1186/s12937-018-0404-4) contains supplementary material, which is available to authorized users.

## Background

Poor diet is one of the most important risk factors for early deaths globally [1]. While public health experts have identified food environments as a driver of poor diet [2–4], they also hold great potential to reduce obesity, non-communicable diseases, and their inequalities [5]. Food environments which can influence eating behaviour include the number, type, location, and accessibility of food outlets present in a community; and the within-store characteristics that can influence food selection [6] including the marketing mix of product, price, placement, and promotion, as well as provision of nutrition information [7]. The term ‘retail food environment’ is also used when referring to supermarkets and other food retail outlets [5].

In Australia, supermarkets are the dominant retail food environment (63% of total food expenditure in 2012-13) [8], and the sector is highly concentrated with the two largest chains accounting for 70% of grocery sales [9]. This is one of the highest levels of supermarket concentration globally [10]. Concentration of grocery sales has taken place in other developed countries [11] including Austria, Canada, Denmark, Germany, France, Spain, and the United Kingdom (UK) [10]. Australian supermarkets hold a powerful position as primary gatekeepers of the food system [12]. They impact public health nutrition by influencing availability, affordability, accessibility, and sustainability of healthy foods [12]. Supermarkets decide the product assortment available, price, promotions, placement of products into aisles, and shelf location [13]. Australian research identified less than half of the packaged foods commonly available in supermarkets were healthy [14].

The power of supermarket chains extends beyond retailing into manufacture, with the introduction of supermarket own brand foods [12]. Supermarket own brand foods (also known as private label, in-house brand, store brand, retailer brand, or home brand) are owned by retailers, wholesalers or distributors and are sold privately in their own stores [15]. They are widely available in Australian supermarkets and around the world [16, 17]. There is wide acceptance of supermarket own brands [18] and they are predicted to reach 35% of Australian grocery sales by 2020 [9]. The highest proportion of supermarket own brand products are found in the UK, Spain and Switzerland where they account for 40-45% of national grocery sales [19]. Supermarkets have control over own brand products, and can determine the choice of ingredients and nutritional content [20], which presents an opportunity for public health professionals to work with supermarkets to improve the nutritional quality of the food supply [7]. However, to date few studies have examined the availability, nutritional quality, price, placement or promotion of supermarket own brand foods in Australia, or elsewhere.

Development of own brand foods is a marketing strategy used by supermarkets to meet a range of objectives which vary according to the product or category. Globally, supermarket own brands have been most successful in high-purchase categories such as bread, milk and eggs; and the categories where consumers perceive little difference when compared with branded products (e.g. canned vegetables) [19]. Supermarket own brands have evolved over time, and now dominate new product launches, aiming to meet consumer demands for convenience and ready-prepared foods [21].

Assessment of the nutritional quality of supermarket own brands has found inconsistent results. Australian research comparing the nutritional quality of supermarket own brands to branded products concluded they could not be described as nutritionally inferior [22], while a more recent study found the mean sodium content was 17% lower compared to branded products from the same categories [23]. A Dutch study found there was no nutritional difference between supermarket own brand foods and branded foods, apart from for sodium where the branded foods contained significantly less [24]. Studies in the UK [25], Spain [26], and Ireland [27] have found no difference in nutritional content between supermarket own brand products and the branded equivalent.

Very little research has investigated the provision of nutrition information on supermarket own brand foods. One Australian study found the only products consistently following the food industry’s voluntary front-of-pack labelling guidelines [28] were supermarket own brands [29].

Supermarket own brand foods will inevitably displace some branded products. Therefore, assessment of the nutritional quality of supermarket own brand foods is needed to enable public health professionals to provide sound advice on their place in the diet.

Australian research shows a significant cost saving for consumers who purchase supermarket own brand foods, making them an appealing option for the budget-conscious. The Food Access and Costs Survey in Western Australia (WA) found that the price of the 2013 *Healthy Food Access Basket* was lower when supermarket own brand products replaced the branded equivalents [30]. The biggest cost savings were for breads and cereals (16%) and dairy (13%) due to the availability of supermarket own brand options in these categories [30]. Supermarket own brand products in the Netherlands [24] and France [31] were also significantly cheaper than the branded equivalent. A UK study found supermarket own brand foods provided consumers with better ‘value for money’, a measure which combined price and nutritional quality [25]. It is important to continue to monitor the price incentive offered by supermarkets to consumers to purchase own brand foods.

To date, no studies have been identified that investigate the placement or promotion of supermarket own brand foods in retail food environments. Australian studies of the placement and promotion of snack foods have highlighted public health issues relating to promotion of foods to children [32, 33] and the prominence given to foods classified as ‘discretionary’ [34, 35]. Given the increasing prominence of supermarket own brand foods, the lack of investigation regarding their contribution to these public health issues is an important gap in knowledge.

A number of survey instruments have been developed to assess and compare retail food environments within supermarkets [36, 37]. A systematic review of available measures recommended that researchers select an existing quality assessed tool where possible [38], and the survey instrument needs to reflect the purpose of the assessment [37]. The widely used United States (US) developed Nutrition Environment Measures Survey in Stores assesses availability of specified healthy options, price and quality [39]. The UK Consumer Nutrition Environment Assessment Tool measures healthfulness of supermarkets, including product variety, price, promotion, shelf placement, store placement, quality, healthier alternatives, nutrition information, and single fruit sale [40]. The US-developed ‘GroPromo’ tool measures product placement and promotion [41]. In Australia, the triennial Food Access and Costs Survey monitors the cost, variety, fresh food quality, availability and nutrition content of 430 foods in stores throughout WA [42]. What is missing is a comprehensive assessment tool that includes the full marketing mix (i.e. product, price, placement, promotion) and describes the contribution of supermarket own brand foods to the healthfulness of retail food environments [7]. The overarching research question this study aims to address is: What is the extent and nature of supermarket own brand foods in Australia?

## Methods/Design

### Study aim

Supermarkets have access to a wealth of information to inform business strategy that directly influences consumer purchasing behaviour and food choice. This information is not readily available to researchers and policy makers. A better understanding of the marketing techniques used by supermarkets within stores to influence consumer purchases of own brands is needed. The aim of this protocol is to describe the methods developed to examine the availability, nutritional quality, price, placement and promotion of supermarket own brand foods within Australian supermarkets.

This study is unique as it aims to investigate all aspects of within-store retail food environments (i.e. product, price, placement, promotion) and address the contribution of supermarket own brands. This protocol could be used to assess supermarket own brand foods in other countries, or to assess the contribution of selected products or brands within retail food environments. It will enable researchers to identify supermarket own brand marketing practices of public health concern, and opportunities for interventions to improve the contribution of own brands to healthful retail food environments in Australia.

The Standard Protocol Items: Recommendations for Interventions Trials (SPIRIT) checklist [43] was used to guide this study protocol, adapted to accommodate the observational study design (Additional file 1: Table S1).

### Setting

#### Selecting supermarkets

One of each major supermarket chain in WA, i.e. Coles Supermarkets Australia Pty Ltd (Coles), Woolworths Supermarkets (Woolworths), and IGA Supermarkets (IGA), were selected. Woolworths and Coles account for 70% of supermarket sales in Australia [9], and are managed from central support offices to maintain general consistency. IGA supermarkets are a heterogeneous mix of store formats owned and operated independently which contribute a low overall share of grocery sales, but represent over 50% of stores in WA [30]. Aldi was excluded from this audit due to the limited range of all products sold compared to the large supermarket chains [44].

Selected supermarkets were conveniently located in Perth, WA. The outlets were selected on the basis of being ‘optimised’ supermarkets, i.e. they were large chain supermarkets with an increased likelihood of stocking most of the own brand product range, and the most up-to-date layouts and displays. The selected Woolworths ‘next generation’ store had been recently extensively refurbished [45]. The selected IGA was an ‘IGA store of the year’ for WA. The selected Coles was the nearest large store to the parent company Wesfarmers’ offices in Perth. These stores should therefore provide good representation of how the supermarket chains would like their stores to look, with well stocked shelves and visually appealing displays.

Each of the supermarket chains was contacted to request assistance in identifying supermarket own brand foods and non-alcoholic beverages (referred to as food hereon in). One supermarket provided detailed information of the own brand product range along with ingredients and nutrition information. Another supermarket chain provided a list of the top selling own brand products and the third supermarket chain declined to provide any information. Permission to conduct the audits was also requested, and support was given by each of the supermarket chains. Final permission was sought from the store manager of the selected supermarkets prior to and during the time of the audits.

#### Identifying supermarket own brand products

Supermarket own brand products were identified as those products carrying the supermarket’s branding on the front-of-pack. ‘Phantom brands’ are owned by supermarket chains but made to appear as if they are not associated with them [46]. Due to lack of association with the supermarket chain on the front-of-pack, it is very difficult to identify these products. Therefore, this study only included the brands that were clearly identified on front-of-pack as owned by supermarkets. Online shopping websites were used to generate product lists to assist with identifying supermarket own brand products in two of the supermarket audits. The third supermarket did not provide this information online.

All supermarket own brand foods present in the three selected supermarkets were audited, including packaged foods and pre-packed fresh products such as fruits, vegetables and meat that carried a supermarket own brand on the label. Forty-three supermarket own brands were identified across the three supermarket chains, the main ones were: Coles, Black & Gold, Community Co., Woolworths, Woolworths Select, and Macro.

#### Identifying retail food environments attributes that can influence food selection

The within-store marketing mix of product, price, promotion and placement were classified into 13 attributes including: (a) product availability and quality; (b) product assortment; (c) design of products and packaging; (d) nutritional quality; (e) provision of supermarket own brand products; (f) pricing strategy; (g) price sensitivity and elasticity; (h) price promotions; (i) in-store location; (j) shelf location; (k) health messages; (l) promotions targeting children; (m) other promotions, adapted from the work of Glanz *et al.* [6, 7]. Information relating to 12 of the 13 attributes were collected in the audit. One attribute, price elasticity, which examines the impact of changes in price on consumer buying behaviour, was not measured as it cannot be collected via a store audit.

### Study design

#### Information audited

The following information was collected during supermarket audits for all own brand food products present:

- Front-of-pack product information including own brand name, product name, product description, pack weight, whether the pack was a multi-pack;
- Design of packaging and label including identification of the country of origin (e.g. Australia made triangle), attributes related to value and convenience;
- Shelf-edge label information including whether it displayed kilojoules, the standard selling price, promoted price, promotion details (e.g. multi-buy, discount, everyday low pricing);
- Placement of the product, including where it was located within the store, on shelf, and the prominence it was given (e.g. using ends of aisles, or placing products at eye level);
- Promotion on the front-of-pack, including presence of supplementary nutrition information (i.e. Health Star Ratings [47] or Daily Intake Guide [28]), nutrition claims, health claims, health marketing techniques, promoting products to children [48], and consumer values issues (e.g. statements and claims about suitability for special diets or animal welfare) [49].

Other sides of own brand packaging, including the back-of-pack, were not collected during the supermarket audits due to time constraints. Back-of-pack information typically includes the barcode, ingredients list, nutrition information panel, and allergen declaration.

#### In-store photography

Photographic images were taken to record the product attributes as quickly as possible as there are constant changes taking place in supermarkets: products are deleted, new products are launched, prices change, price promotions are implemented on a weekly basis, and there are seasonal changes in availability of fresh produce and other products (e.g. Easter eggs). Photographic methods enabled quick data collection and have been used to assess and monitor packaged foods in supermarkets previously. Photographic audits are less expensive and a more efficient way of collecting product information within supermarkets, compared to purchasing products or completing paper-based surveys [50].

#### Data collection

Two researchers visited each store together, during a 3-week period commencing in February 2017. This date was selected to avoid the changes that occur in supermarkets during the Christmas and Australian summer holiday period, and prior to Easter. Data collection took a total of eight days; three days in two stores, and two days in the final store. Audits commenced upon store opening in the morning to minimise disruption to the stores, and were ceased if the stores became too busy to photograph products unobtrusively.

For quality control, each of the stores was divided into product zones based on the physical location of products (e.g. fresh produce, frozen food) and each researcher photographed the zones they were designated. Photographs were taken to show the location of the product display within the store, the location of products on each of the shelves, and the use of any display materials such as shelf-edge labelling or large signs. The front-of-pack and shelf-edge label for each supermarket own brand product identified was photographed. For products that were not available, photographs were taken of the empty product space and shelf-edge label and products were photographed at a later date, if present during the audit period. Products that were not present throughout the audit period were not included in this study.

At regular intervals both researchers walked through the zones together to check that all products had been identified and photographed. This was done by referring to the product lists generated prior to conducting the supermarket audits, and by examining the products available. Any missed products were photographed during this process. Breaks were also taken at regular intervals to upload and back-up photographs to a laptop computer. At the end of each day photographs were reviewed for legibility, and any illegible photographs that could not be used were listed and retaken the following day. Photographs were date and time tagged by the devices used.

Supermarket own brand ready-to-eat or ready-to-heat mixed food products that require refrigeration, for example chilled ready-meals, were photographed in-store as part of standard data collection, and then purchased to enable further photographic collection of information provided on the back and sides of the packages.

Purchased products were photographed in a food sensory laboratory at Curtin University. Each chilled convenience product was assigned a code, which was visible in the photographs and recorded on a spread sheet. This code ensured easy identification of the product and associated supermarket, and prevention of product misrecognition during data extraction, particularly for the back of pack images. To prevent food waste, the chilled, un-opened products were delivered to a local food charity to redistribute.

### Data management

#### Database and data extraction

An electronic computer filing system was established for each of the supermarkets, with folders for each of the 18 product zones, or food groups, identified in the supermarkets. Product and display photographs were filed accordingly.

A database was constructed to enable systematic entry of store audit photographs information using Microsoft Excel (Version 2013, Redmond, Washington, USA). Each supermarket was assigned a separate spreadsheet, with separate worksheets created for each of the 18 product zones, or food groups, (e.g. frozen food). Product groups were identified for each zone, so that products could be allocated to a group (e.g. ice cream). Pre-coded responses were established for each of the columns for data entry, to enable consistent classification across supermarkets, product areas, and between researchers. Free text was permitted for product name, product description, price, promoted price, shelf position details, location prominence details, and columns for details relating to each of the promotions data. The researchers who conducted the supermarket audits completed data entry.

The first product zone, or food group, for the first supermarket was piloted to ensure all necessary information was collected, and establish any final changes needed to the pre-coded responses. After completing data entry, both researchers reviewed the data and changes were implemented by the first author as required to ensure consistency of approach. Specific procedures for classification of product nutritional quality were developed which are addressed below.

### Assessment procedures

#### Front-of-pack product information

Information was extracted from the supermarket own brand front-of-pack photographic images including: product name, product description, whether the product was a pack containing multiple units (i.e. multipack), and the pack weight or volume and entered directly into the database. Products were assigned to one of 18 food groups, and one of 130 product groups (see Additional file 2: Table S2).

#### Shelf-edge label information

Information was extracted from the shelf-edge label photographic images including: the standard price per pack, promoted price per pack, price promotion details, and whether kilojoule labelling was present, and entered directly into the database. Price per 100 grams or 100 millilitres, and price per item for multipacks were calculated. Price promotions were classified according to the key message used including: half price, every day, locked down low prices, special, value, multi-buy offers, and percentage off discounts.

#### Design of label and packaging

In Australia, packaged foods must carry a statement identifying the country where the food was made, produced or grown, or manufactured or packaged [51]. The audit collected the design attributes used on the front-of-pack and shelf-edge-labels to identify foods as Australian including: Australian flag, map or outline of Australia, the Southern Cross stars, the Australian made triangle, the updated Australian made triangle with a ruler depicting the proportion of ingredients that are Australian, or stating Australia in the product title or description.

Supermarket own brand foods initially provided a low quality unbranded alternative to branded products at a lower price [52]. Techniques used on supermarket own brands to communicate value for money were identified on the photographic images including: use of plain packaging or few colours, price marked packs, use of promotional stickers, and using words to indicate value.

Techniques used on supermarket own brands to demonstrate convenience were identified in photographs including: single-serve packs, packaging with cutlery included, packaging that reveals ready-to-eat or ready-to-heat foods that require little effort to prepare, foods presented in convenient packaging formats such as oven-ready trays or microwavable or resealable containers, and words used to convey the speed of preparation.

#### Placement of the product

When shoppers notice a product, they are more likely to buy it [53] therefore high footfall locations within the store such as ends-of-aisles and the entrance can impact consumer purchases. For this audit, the in-store location was recorded including whether the product featured on a special display.

Products are also more likely to be purchased when placed in prominent shelf positions such as at eye level [54]. An existing audit tool, the Consumer Nutrition Environment Assessment Tool, included criteria to identify the most prominent shelf at eye level, the least prominent at the bottom of the display, and other shelves classified as less prominent [40]. This current protocol adapted the classification to include the range of display units present in the supermarkets, such as market-style bins and refrigerated barges, identifying the most prominent, least prominent, and less prominent shelf positions.

For prominence, a number of techniques were identified during the audits, including highlighting the product location with signage such as shelf stripping or signs, displaying the own brand products together creating an ‘own brand block’, displaying the same own brand product in more than one location, and placing own brand products adjacent to the higher profile branded equivalent.

#### Product promotion on the front-of-pack

**Fig. 1:**
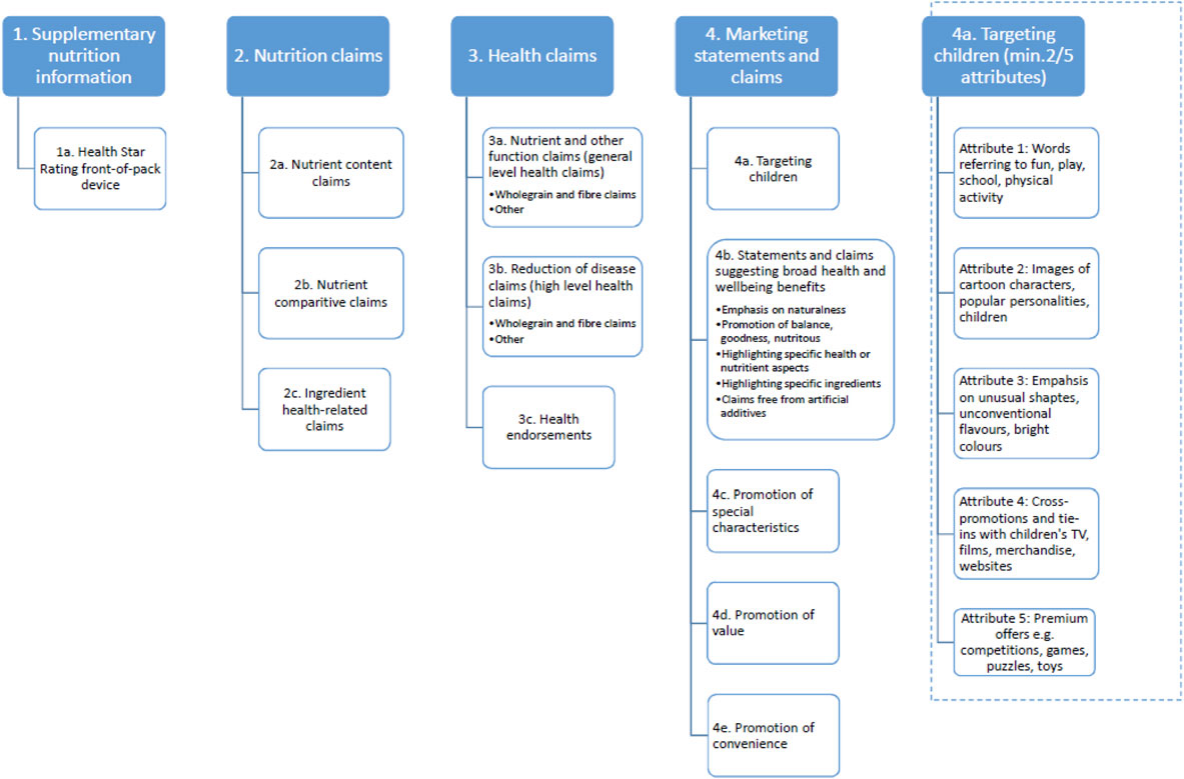
Taxonomy of nutrition and health related packaging information [48]. Adapted from the INFORMAS food labelling taxonomy [77], Mayhew *et al.*’s definitions of marketing techniques promoting health and wellbeing [78]; and Mehta and colleagues’ work defining food packaging targeting children [79]

A taxonomy of nutrition and health related packaging information to identify supplementary nutrition information, nutrition claims, health claims, and marketing statements and claims has previously been constructed [48] and was utilised for this study (Fig. 1). Three additional marketing techniques used by supermarket own brands to appeal to children were identified in this audit: mini or child portioned packs, reference to children or ‘kids’ in the product name or branding, and placement of a supermarket own brand product adjacent to a similar branded child-targeted product.

Supplementary nutrition information on the front-of-pack is voluntary in Australia. There are two commonly applied systems: the government-led Health Star Ratings (HSR) and the food industry-led Daily Intake Guide (DIG). The HSR was designed to be applied to packaged processed foods and uses an algorithm to assign each product a score from ½ to 5 health stars, with 5 stars indicating the healthiest choice [47]. The product can feature one of three versions of the device which include (a) the HSR only, (b) the HSR plus kilojoules per 100 grams, or (c) the HSR plus kilojoules, saturated fat, sugars, sodium per 100 grams and an optional nutrient [55]. The DIG provides nutrition information on the front-of-pack. There are two versions which can be applied: (a) the DIG thumbnail icon displaying kilojoules per serve; and (b) the DIG preferred format of kilojoules, fat, saturated fat, sugars and sodium per serve [56]. This audit identified presence of the following from the front-of-pack photographs: HSR only, HSR plus kilojoules, HSR plus kilojoules and nutrients, the DIG thumbnail, the DIG with nutrients, a nutrition information panel, or an ingredients list.

An Australian independent review of food labelling law and policy identified ‘consumer values issues’ as issues of importance to consumers but not directly affecting health [49]. Communication of consumer values issues on the front-of-pack were identified in this audit including: organic food, food containing no MSG, beef with no growth hormones, and food containing no artificial colours or flavours.

Supermarket corporate social responsibility (CSR) statements made on the front-of-pack were also identified and a free text column provided to note the details including: commitments to sustainable fishing practices and supporting local farmers.

#### Nutritional quality

Products were assessed for nutritional quality using the front-of-pack information collected during the audits. The HSR was noted as provided on pack, and it was not calculated for products that didn’t display the device. Products were classified into food groups consistent with the Australian Guide to Healthy Eating (AGTHE) [57], the NOVA classification of level of food processing [58], and the expanded classification of level of food processing developed by Poti *et.al.* (2015) which includes three levels of convenience [59].

The NOVA classifications were referred to for classification of the supermarket own brand products [58] and the Poti *et al.* category definitions and criteria were used to classify foods based on the level of industrial processing and the amount of preparation required by the consumer [59].

Classifying foods according to the AGTHE proved more problematic as the examples provided in the Educators Guide [60] are limited to whole foods, not meals or mixed foods, and provide overarching principles that can be applied to dietary analysis more easily than packaged food categorisation. The Australian Bureau of Statistics (ABS) established principles for identifying ‘discretionary foods’, not essential for a healthy diet, in order to conduct analysis of the national food and health survey [61]. This method was adapted as there were many ready-to-eat products in the audit which were not addressed by the ABS criteria. A decision tree was constructed to enable categorisation of products in accordance with the principles of the AGTHE, with the addition of two new groupings: ‘Mixed products using mainly five food group foods’, and ‘Mixed products high in fat, salt or sugar’ (Table 1).

**Table 1:** Procedure to classify foods consistent with the Australian Guide to Healthy Eating

Question	Details	If yes…	If no or unsure…
Q1. Is the product easily identifiable as a five food group food, or water?	Vegetables - All fresh, frozen, canned and dried, but not fried	Classify into the appropriate food group	Go to Q2
	Fruit - All fresh, frozen, canned, dried, and fruit juice		
	Grains - Whole and rolled grains, flour, bread, pasta, noodles, breakfast cereals, including refined and whole grain varieties		
	Lean meat, fish, and alternatives - All fresh, frozen and canned meat, poultry and fish; salt and fat reduced sausages; eggs, tofu, nuts and nut spreads, legumes, seeds		
	Milk, yoghurt, cheese, and alternatives - Fresh, dried, evaporated or UHT milk, yoghurt, all cheese, and calcium-enriched alternatives		
	Water		
Q2. Is the product easily identifiable as a discretionary food, using the examples provided in the Eat for Health Educators Guide?	Foods with higher added sugars - energy drinks, fruit drinks, honey, jams, marmalade, some sauces, sports drinks, sugar, confectionery, soft drinks, cordials, sweetened waters, iced tea, syrups	Classify as discretionary	Go to Q3
	Foods with higher saturated fat - bacon, ham, butter, cream, ghee, some tacos/nachos/enchiladas, commercially fried foods, commercial burgers, crisps, extruded snacks, dairy blends, frankfurts, chips, meat pie, pasties, pastry, pizza, processed meat, quiche, salami, mettwurst, sausages, some crackers, some sauces, spring roll		
	Foods with higher saturated fat and added sugars - biscuits, cakes, chocolate, chocolate bars, dessert style custards, doughnuts, iced buns, ice cream, muesli bars, puddings, slices, some confectionery, some sauces, muffins, pastries, pies, crumbles		
	Foods with high salt - marinades and sauces e.g. fish sauce, soy sauce; salty snack foods; spreads e.g. Vegemite; savoury biscuits		
Q3. Do the ABS principles for identifying discretionary foods identify this food as discretionary?	All milk drinks including flavoured milk	Classify as milk, yogurt, cheese and alternatives	Go to Q4
	All soft drinks including those with intense sweeteners	Classify as discretionary	
	All fruit drinks other than fruit juices		
	Tea or coffee with added sugar		
	Breakfast cereals without added fruit > 30g sugar/100g		
	Breakfast cereals with added fruit > 35g sugar/100g		
	All dry soup mixes		
	Mixed dishes containing grains e.g. sandwiches, burgers, wraps, sushi, pizza >5g saturated fat/100g	Classify as ‘mixed product high in fat salt or sugar’	
Q4. Does the product contain any of the following: added saturated fat, added salt, or added sugar?	Added saturated fat e.g. butter, cream, coconut milk/cream, mayonnaise	Go to Q5	Classify as 'mixed product using mainly five food group foods'
	Added salt e.g. marinades, soy/fish sauce, stock/bouillon		
	Added sugar or other sweeteners e.g. honey, syrups		
Q5. Does the nutrition content of the product meet any of the following criteria from the Eat for Health Educators Guide?	-- total fat > 10g per 100g	Classify as discretionary or 'mixed product high in fat salt or sugar'	Go to Q6
	-- saturated fat > 3g per 100g		
	-- total sugar > 15g per 100g		
	-- sodium > 400mg per 100g		
Q6. Is there enough information provided to classify the product as five food group foods or mixed product using mainly core foods?	For products where only front-of-pack information is available, products will be classified as discretionary/ mixed product high in fat salt or sugar unless there is sufficient information to classify it as five food group food/ mixed product using mainly five food group foods	Classify into the appropriate food group, or as 'mixed product using mainly five food group foods'	Classify as discretionary or 'mixed product high in fat salt or sugar'

### Data analysis

Approximately 20,000 photographic images were collected for 3940 supermarket own brand foods in the audit, and details recorded in the database. There were 1812 supermarket own brand foods present in the Woolworths store, 1731 supermarket own brand foods in the Coles store, and 397 supermarket own brand foods in the IGA store. Research questions relating to 12 of the 13 attributes of within-store retail food environments have been identified (Table 2). All data will be entered into SPSS for Windows (Version 24, Released 2016, IBM Corp., USA) and summarised using descriptive statistics, frequencies and presented graphically using bar charts.

**Table 2:** Relationship between within-store retail food environment attributes, research questions, and data collection for the Supermarket Nutrition Environment Assessment Tool – Supermarket Own Brands

Attribute^a^	Research questions	Data required
Product		
(a) Product availability and quality	• What is the availability of healthy and unhealthy own brand foods in Australian supermarkets? • What proportion of supermarket own brand foods are Australian made?	Supermarket own brand name, product name, product description, pack size, pack weight, price, price promotion, Australia made logo, Australia included in product title or description
(b) Product assortment	• How many supermarket own brand foods are available? • How much variety of supermarket own brand foods is available i.e. breadth of choice across categories and depth of choice within each category, particularly in ready-to-eat foods?	Products assigned to one of 18 food groups, and 131 product groups
(c) Design of products and packaging	• How many own brands are used by Australian supermakets? • What supplementary nutrition information is made available on front-of-pack of supermarket own brands? • What is the prevalence of messages promoting value or convenience on supermarket own brand foods?	Supermarket own brands packaging design techniques including words/ colours/ images promoting value or convenience, front-of-pack supplementary nutrition information
(d) Nutritional quality	• What supermarket own brand foods are available in each of the AGTHE food groups? • How do supermarket own brand foods rate using the HSR system? • What is the prevalence of healthy lines of supermarket own brand foods? • How are supermarket own brand foods categorised using the NOVA system?	Supermarket own brands to be classified using the AGTHE and NOVA using front-of-pack information only; HSR to be recorded from front-of-pack
(e) Provision of supermarket own brand products	• What is the prevalence of supermarket own brand ethically sourced foods? • What is the prevalence of supermarket own brand convenience foods?	Supermarket own brand statements and logos relating to ethical food standards; messages and design techniques relating to convenience
Price		
(f) Pricing strategy	• How does the price of healthy supermarket own brand foods compare with unhealthy own brand foods?	Analysis using price and nutritional quality data
(h) Price promotions	• How are supermarket own brand foods promoted using price? For example, using price reductions, multi-buy offers, everyday low pricing, coupons, and price marked packs. • How does price promotion of healthy supermarket own brand foods compare with unhealthy own brand foods?	Analysis using supermarket own brands price promotion techniques and nutritional quality data
Placement		
(i) In-store location	• Where are supermarket own brand foods physically located within stores? For example, are any at the ends-of-aisles, at checkouts, in island dump bins? • What is the prevalence of co-locating supermarket own brand foods adjacent to the branded equivalent?	Supermarket own brands physical location in store, including whether on the perimeter of the store, or the aisle
(j) Shelf location	• How prominently located are supermarket own brand foods? • How is supermarket signage or décor used to give supermarket own brands prominence?	Supermarket own brands prominence in store, including whether in blocks, at eye level, large number of shelf facings, and signage or décor
Promotion		
(k) Health messages	• How is supermarket own brand packaging information classified using a taxonomy of nutrition and health related packaging information? • How are the quality standards applied to supermarket own brand foods communicated to shoppers? • How are the ethical standards applied to own brand foods communicated to shoppers?	Marketing techniques and nutrition and health statements and claims, logos or statements about product quality or quality standards in general, and logos or statements about ethical standards
(l) Promotions targeting children	• What is the prevalence of supermarket own brand foods designed to appeal to children? • What proportion of supermarket own brand products designed to appeal to children can be described as healthy?	Marketing techniques designed to appeal to children (included in the taxonomy above); analysis of the nutritional quality of selected products
(m) Other promotions	• What other techniques are used on supermarket own brand products?	Information from the front-of-pack of supermarket own brands

^a^Attributes adapted from Glanz and colleagues [6, 7]; *AGTHE* is Australian Guide to Healthy Eating; *NOVA* is a classification system based on the level of food processing; *HSR* is the Health Star Rating front-of-pack labelling system

## DISCUSSION

The aim of this protocol was to describe the methods developed to examine the availability, nutritional quality, price, placement and promotion of supermarket own brand foods within Australian supermarkets. This study aimed to investigate all aspects of within-store retail food environments and address the contribution of supermarket own brands.

Supermarket outlets operated by the large chains are managed from central support offices for consistency, but are not homogenous as the products and services may differ by store [62]. The International Network for Food and Obesity Research Monitoring and Action (INFORMAS) recommends monitoring food availability in predominant food environments [5]. The supermarkets selected for this study were ‘optimised’ to reflect the way the chains would like stores to look. This approach was taken so that the study would provide information about a wide selection of supermarket own brand foods, and how they are marketed. Other approaches could be taken for audits, including selecting stores based on the socio-economic profile of the neighbourhood, or level of geographic isolation.

Supermarket own brand products were selected as the focus of this study as little is known about their availability, nutritional quality, price, placement or promotion. In Australia, powerful supermarkets control own brand products [12] and implement corporate social responsibility (CSR) initiatives to manage their impact on the communities where they operate [63, 64]. In a neoliberal political context, whereby government regulation is minimized to promote free trade [65], consumers rely on such voluntary measures to support public health. International examples of supermarket CSR initiatives that impact public health include: banning the sale of energy drinks to children [66]; removing lunchbox-sized sugar sweetened beverages from sale [67]; introducing a supermarket-wide shelf-edge labelling system that identifies healthy foods [68]; and improving the nutritional quality of own brand foods [69, 70]. Interventions in supermarket settings are generally effective in improving food purchasing patterns, and can play a role in protecting public health [71–73]. Therefore, findings from this study will assist researchers in identifying own brand marketing practices of public health concern, and opportunities for interventions to make improvements. Supermarket CSR initiatives will be recommended, for example making targeted changes to own brand foods that can improve the nutritional quality of the food supply [7]. The protocol of this study could be adapted for other countries with high proportions of supermarket own brand products (e.g. Spain, the UK, Switzerland [19]) with results used in a similar way.

This research protocol could also be adapted to understand how supermarkets market other products (e.g. sugar sweetened beverages and energy drinks) or brands (e.g. Nestle), or identify marketing techniques used to appeal to children. The INFORMAS recommendations for advocacy initiatives to promote public health include holding companies, such as food manufacturers and supermarkets, to account for actions that impact public health [74]. This can be done by naming and shaming poor practice, or acknowledging and praising good practice [74]. This advocacy strategy recognises that food companies, including supermarkets, have the collective power to improve food environments and assist consumers to select healthy foods [75]. Adapting this study’s protocol to conduct within-store audits of specific products or brands could assist with identifying marketing practices of concern to public health, as well as CSR initiatives that have had a positive impact.

Existing assessment tools were referred to in the construction of this protocol. However, no tool was identified that evaluated the full marketing mix and nutritional quality of selected products within retail food environments (Table 3). The UK Consumer Nutrition Environment Assessment Tool included criteria to identify the most prominent shelf placement and store placement. The WA Food Access and Costs Survey included key variables for price, promotions, availability, and nutrition content [42]. Previous work on a smaller product sample informed the nutrition and health related data collected [48].

**Table 3:** Within-store retail food environment attributes examined in key survey instruments

Attribute^a^	Nutrition Environment Measures Survey – Stores (NEM-S) [39]	Gro-Promo [41]	Consumer Nutrition Environment Assessment Tool [40]	WA Food Access and Costs Survey (FACS) [30]	Supermarket Nutrition Environment Assessment Tool – Supermarket Own Brands
Product					
Product availability and quality	✓	-	✓	✓	✓
Product assortment	✓	-	✓	-	✓
Design of products and packaging	-	-	-	-	✓
Nutritional quality	✓	-	-	-	✓
Provision of supermarket own brand products	-	-	✓	✓	✓
Price					
Pricing strategy	✓	-	✓	✓	✓
Sensitivity and elasticity	-	-	-	-	-
Price promotions	-	-	✓	✓	✓
Placement					
In-store location	-	✓	✓	-	✓
Shelf location	✓	✓	✓	-	✓
Promotion					
Health messages	-	-	-	-	✓
Promotions targeting children	-	✓	-	-	✓
Other promotions	-	✓	-	-	✓

^a^Attributes adapted from Glanz and colleagues [6, 7]

Assistance was provided by two of the three supermarkets included in this study. The product lists provided were not as helpful as they first seemed. One supermarket chain provided information about all existing own brand products. However, it is unlikely that any supermarket outlet would stock all currently available products. The list included products being phased out, new products not yet launched, and seasonal products that are only available at certain times of the year. Due to the long distances between food producing areas and urban centres in Australia [76] each State or Territory can stock locally produced foods not available elsewhere. Some products were identified as not available by the empty space on the shelf during the audit. When products are not available for more than a few days the space is likely to be filled with other products and the shelf-edge label removed. A second supermarket chain provided a list of top selling products, and similar problems were encountered during the audit. The researchers used the lists provided by supermarkets as guidance to the names of the own brands and the types of categories where products would be present. The product lists generated from the shopping websites were more useful, but did not include all supermarket own brand products present in the stores audited.

Researchers were sensitive to the needs of supermarket staff and customers, and timed the audits to avoid peak shopping times. Use of photographic images proved to be a quick and efficient way of collecting data unobtrusively. Photographs were taken to show the location of the product display within the store, location of products on shelves, use of display materials, and the front-of-pack and shelf-edge label for each supermarket own brand product identified. Regular review of the photographs for legibility was essential, so that gaps in data could be filled during the audits. Whilst the photographic images from the audits were legible, sometimes the angle of a photograph missed an important variable. For example some front-of-pack images showed products displayed in a shelf ready carton where supplementary nutrition information was not visible. To fill these gaps researchers searched for missing packaging information on the supermarket shopping websites, or in a local supermarket.

### Limitations

This protocol has a number of strengths and limitations. The extensive nature of the data collected is likely to provide great insight into the contribution of supermarket own brand foods to the healthfulness of retail food environments in Australia. The study utilised a detailed taxonomy which had already been tested and applied to a smaller sample of products. The protocol may be adapted for use in other countries with high proportions of supermarket own brand foods, or to evaluate the contribution of other significant product groups or brands to within-store retail food environments. The protocol described in this study took place with support from the central office of each supermarket chain, and permission was granted by the store managers. This was despite initial reluctance to allow photography in one of the stores. Without permission from supermarkets for photography, data collection of this scale would not be possible. Even so, due to the large number of products audited there is a possibility that some supermarket own brand foods were missed. The systematic data collection using photographic methods proved to be quick and efficient. Data management of the photographs into a designated electronic filing system was essential and proved effective. However, gaps in information were identified during data extraction and needed to be filled using suitable alternative sources including the supermarket shopping websites. Data collection of branded products was not included in this study protocol, as that was not the purpose of this study. Future within-store audits of supermarket own brand foods could include the branded equivalents to enable analysis of the similarities and differences in the marketing techniques employed.

## Conclusion

This protocol describes the methods developed to examine the availability, nutritional quality, price, placement and promotion of supermarket own brand foods within Australian supermarkets. This is important because Australian supermarkets hold a powerful position as primary gatekeepers of the food system, and consumers rely on their voluntary CSR initiatives to support public health. However, little is known about the availability, nutritional quality, price, placement or promotion of supermarket own brand foods. Existing survey instruments do not comprehensively assess the full marketing mix (i.e. product, price, placement, promotion) or describe the contribution of specific foods, such as supermarket own brand foods, to the healthfulness of retail food environments. Therefore, this protocol describes methods for collecting the data required to assess all aspects of within-store retail food environments using photographic images. Analysis of findings of the 20,000 photographic images for 3940 foods will enable researchers to identify own brand marketing practices of public health concern, and opportunities for interventions to improve the contribution of supermarket own brands to healthful retail food environments in Australia. Supermarket CSR initiatives that can have a positive impact on public health will also be recommended. The study protocol could be adapted for other countries with high proportions of supermarket own brand foods (e.g. Spain, the UK, Switzerland) with results used in a similar way. It could also be adapted to understand how supermarkets market other products (e.g. sugar sweetened beverages and energy drinks) or brands (e.g. Nestle), or identify marketing techniques used to appeal to children. Dissemination of results to public health researchers and policy makers will enable full evaluation of the protocol’s utility.

## Additional files

Additional file 1: **Table S1.** Completed SPIRIT 2013 Checklist: Recommended items to address in a clinical trial protocol and related documents* (DOC 100 kb)

Additional file 2: **Table S2.** Food groups and product groups for classifying supermarket own brand foods (DOCX 15 kb)

## Abbreviations

AGTHE: Australian Guide to Healthy Eating
DIG: Daily Intake Guide
CSR: Corporate Social Responsibility
HSR: Health Star Rating front-of-pack labelling device
WA: Western Australia
