# Supplementary material for: The extent and nature of supermarket own brand foods in Australia: study protocol for describing the contribution of selected products to the healthfulness of food environments
Source: Nutr J. 2018 Oct 25;17:95. doi: 10.1186/s12937-018-0404-4 (PMC6201490; doi:10.1186/s12937-018-0404-4)
Supplement: Supplementary file 2 — Table S2. Food groups and product groups for classifying supermarket own brand foods (DOCX 15 kb) [file 12937_2018_404_MOESM2_ESM.docx]

**Supplementary Table 2: Food groups and product groups for classifying supermarket own brand foods**

| **Food group** | **#1 Bakery and desserts** | **#2 Beverages** | **#3 Chilled convenience** | **#4 Frozen food** | **#5 Dairy** | **#6 Processed meat** | **#7 Prepared meat** | **#8 Processed fish** | **#9 Processed fruit, veg, legumes** | **#10 Rice, pasta, noodles, cereal, other grains** | **#11 Snacks, crisps, nuts** | **#12 Dried fruit** | **#13 Confectionery** | **#14 Soups, sauces, condiments** | **#15 Baby** | **#16 Baking ingredients** | **#17 Prepacked fresh food** | **#18 Other grocery** |
| --- | --- | --- | --- | --- | --- | --- | --- | --- | --- | --- | --- | --- | --- | --- | --- | --- | --- | --- |
| **Product group** | Biscuits and slices | Carbonated drinks | Chilled antipasto and dips | Frozen baking ingredients | Butter and margarine | Bacon/ ham | Prepared beef | Canned tuna | Canned/jar veg | Breakfast cereals | Crisps and chips | Dried fruit | Confectionery | Canned soup | Baby food | Baking ingredients | Prepacked beef | Coffee |
|  | Bread and alternatives | Chilled juice | Chilled dressed salads | Frozen desserts | Cheese | Burgers/ rissoles | Prepared chicken | Canned salmon | Canned fruit | Canned rice | Nuts | Dried fruit snacks | Chocolate | Condiments | Infant formula | Cake mixes | Prepacked chicken | Herbs and spices |
|  | Cakes and pastries | Cordial | Chilled garlic bread and naan | Frozen fish | Dairy desserts | Canned meat | Prepared lamb | Smoked fish | Canned legumes | Legumes | Nut snacks | Fruit and nut mix | Chocolate coated confectionery | Gravy |  | Biscuit mixes | Prepacked fish | Hot chocolate |
|  | Desserts | Fruit drink | Chilled meals | Frozen fruit | Dairy snacks | Chilled sauces and marinades | Prepared pork | Other fish | Canned pasta in sauce | Instant meals | Popcorn |  |  | Packet soup |  | Pancake mixes | Prepacked fruit | Jams and spreads |
|  | Ice cream cones and toppings | Long-life juice | Chilled pasta and sauce | Frozen hand held ices | Eggs | Sausages and meatballs |  |  | Dehydrated veg | Liquid breakfast | Salsa |  |  | Sauces |  | Cake toppings | Prepacked herbs/ seasoning | Long-life meals |
|  | Savoury biscuits/ snacks | Water | Chilled pizza | Frozen meals | Gourmet cheese | Cured meat |  |  | Vegetable pouch | Other grains and seeds | Snack bars/ snack pots |  |  | Soup pouch |  | Cooking chocolate | Prepacked lamb | Oil |
|  |  |  | Chilled quiche and pies | Frozen meat | Long-life milk and cream | Sliced/ processed meat |  |  | Fruit cup/ fruit tub | Pasta |  |  |  | Stock |  | Custard | Prepacked pork | Salad dressing |
|  |  |  | Chilled soup | Frozen party food | Milk and cream | Other |  |  | Fruit pouch | Rice |  |  |  |  |  | Flour | Prepacked mixed meat | Salt and seasoning |
|  |  |  | Prepared vegetables | Frozen pizza | Yogurt |  |  |  |  |  |  |  |  |  |  | Sugar and syrups | Prepacked salads | Sugar and sweeteners |
|  |  |  | Vegetarian | Frozen potatoes |  |  |  |  |  |  |  |  |  |  |  |  | Prepacked veg | Tea |
|  |  |  | Sauces and salad dressing | Frozen vegetables |  |  |  |  |  |  |  |  |  |  |  |  |  | Vinegar |
|  |  |  | Salad kits and bowls | Ice cream |  |  |  |  |  |  |  |  |  |  |  |  |  | Other grocery |
